# *Acorus tatarinowii* Schott: A Review of Its Botany, Traditional Uses, Phytochemistry, and Pharmacology

**DOI:** 10.3390/molecules28114525

**Published:** 2023-06-02

**Authors:** Meng Wang, Hai-Peng Tang, Shuang Wang, Wen-Jing Hu, Jia-Yan Li, Ai-Qi Yu, Qian-Xiang Bai, Bing-You Yang, Hai-Xue Kuang

**Affiliations:** Key Laboratory of Basic and Application Research of Beiyao, Ministry of Education, Heilongjiang University of Chinese Medicine, Harbin 150040, China

**Keywords:** *Acorus tatarinowii* Schott, botany, traditional uses, phytochemistry, pharmacology

## Abstract

*Acorus tatarinowii* Schott (*A. tatarinowii*) is a natural medicinal plant. It plays an indispensable role in the treatment of diseases by the empirical medicine system and has achieved remarkable curative effects. *A. tatarinowii* is often used to treat various diseases, such as depression, epilepsy, fever, dizziness, heartache, stomachache, etc. More than 160 compounds of different structural types have been identified in *A. tatarinowii*, including phenylpropanoids, terpenoids, lignans, flavonoids, alkaloids, amides, and organic acids. These bioactive ingredients make *A. tatarinowii* remarkable for its pharmacological effects, including antidepressant, antiepileptic, anticonvulsant, antianxiety, neuroprotective, antifatigue, and antifungal effects, improving Alzheimer’s disease, and so on. It is noteworthy that *A. tatarinowii* has been widely used in the treatment of brain diseases and nervous system diseases and has achieved satisfactory therapeutic effects. This review focused on the research publications of *A. tatarinowii* and aimed to summarize the advances in the botany, traditional uses, phytochemistry, and pharmacology, which will provide a reference for further studies and applications of *A. tatarinowii*.

## 1. Introduction

*Acorus tatarinowii* Schott. (*A. tatarinowii*) is a common perennial herbaceous plant [1]. It is mainly distributed from northern temperate to subtropical regions, especially in China, Japan, India, Thailand, and Korea [2]. *A. tatarinowii* is one of the most widely distributed and frequently used natural medicinal plants from the genus *Acorus* and has a long documented history of medicinal use in the empirical medical system. *A. tatarinowii* first appeared as a traditional Chinese medicine (TCM) in the earliest Chinese medicinal classic work Shennong’s Classic of Materia Medica (written more than 2000 years ago during the Han Dynasty). It is widely used in folk medicine for treating complex and difficult ailments as well as some serious diseases and has achieved remarkable therapeutic effects. It was included for the first time in the 1963 edition of the Pharmacopoeia of the People’s Republic of China [3] as a TCM in clinical use and was continuously included until the latest 2020 edition [4]. Dried rhizomes are the main *A. tatarinowii* medicinal parts, and these have been commonly used alone or combined with other TCM in China to treat stroke, dementia, depression, seizure, and mental disorders for centuries [5]. Many Chinese medicinal formulae containing *A. tatarinowii* have been widely used in clinical practice. At the same time, many commercial Chinese patented medicinal products containing *A. tatarinowii* are circulating in the market to treat specific diseases. Moreover, certain active ingredients are extracted from *A. tatarinowii* as pharmaceutical raw materials [6]. As a medicinal plant, *A. tatarinowii* has significantly contributed to people’s health and the traditional medical systems.

Over the past few decades, *A. tatarinowii* has attracted increasing interest as an important medicinal plant from both researchers in natural medicine and pharmaceutical institutions. Significant progress in the isolation and identification of *A. tatarinowii* active constituents has been made. *A. tatarinowii* contains many phytochemical components with diverse structures and different activities. Thus far, more than 160 components have been identified and characterized. They mainly include phenylpropanoids, terpenoids, lignans, flavonoids, alkaloids, amides, organic acids, and others. Modern pharmacological studies have shown that these chemical components have potent properties, such as antidepressant, antiepileptic, anticonvulsant, antianxiety, antifatigue, and antifungal properties, and they have been shown to improve Alzheimer’s disease (AD) [7,8]. It is worth noting that *A. tatarinowii* has shown potent neuroprotective effects. *A. tatarinowii* can reduce brain nerve injury by regulating neurotransmitter levels and improving blood circulation in the brain. It offers good protection for the brain’s nervous system. Whether used alone or as a prescription, *A. tatarinowii* is an important and indispensable herb to treat depression and is used in the TCM treatment system [9]. However, some clinical observations have shown that the active ingredients of *A. tatarinowii* have potential toxicity. Therefore, it is necessary to be cautious in using *A. tatarinowii* as a treatment method, strictly control the dose of *A. tatarinowii*, and better protect people’s health [10]. If it is disturbed and stimulated by the external environment, it will aggravate the poisoning condition. Therefore, safety measures and comprehensive research should be carried out in the future [11,12]. The exploitation and TCM applications in the prevention and treatment of various diseases are gradually growing due to the in-depth study of TCM. Thus, research on *A. tatarinowii* is becoming increasingly necessary [13].

Research on *A. tatarinowii* has advanced significantly due to recent international growth in TCM recognition and contemporary scientific and technical advancements. Reviewing the existing and available literature, it was found that although a large number of studies have been carried out on *A. tatarinowii*, they mainly focus on a single aspect of its phytochemistry or pharmacology. However, there is still a lack of a comprehensive review specifically for *A. tatarinowii*. Therefore, it is very important and necessary to conduct a comprehensive review of the research progress on *A. tatarinowii* in recent years. This is the first up-to-date review of *A. tatarinowii* research developments which includes its botany, traditional uses, phytochemistry, and pharmacology. It offers a review of *A. tatarinowii* research, points out gaps in existing research, and suggests new areas for investigation. The authors hope this review will inspire new research on the pharmacological effects and processes behind *A. tatarinowii* therapeutic effects and provide researchers with a wider perspective and fresh ideas for studying the plant’s present and prospective uses.

## 2. Botany

**Figure 1:**
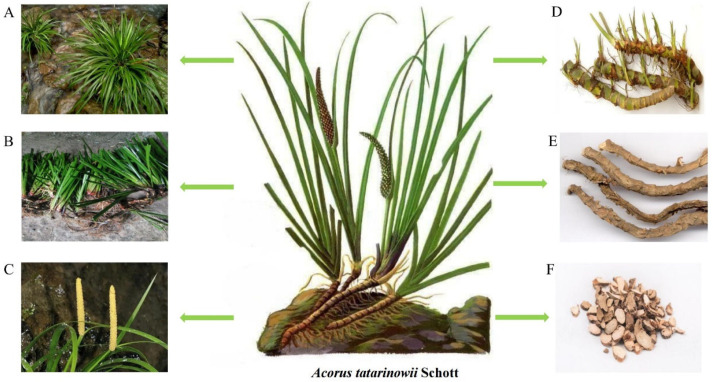
Plant morphology of *A. tatarinowii*. (**A**) Whole plants, (**B**) leaves, (**C**) inflorescences, (**D**) rhizomes, (**E**) dry rhizomes, and (**F**) Chinese herbal pieces.

*A. tatarinowii* is a semi-evergreen perennial hairless plant. It usually grows in creeks, ponds, and other humid environments below 2600 m. According to the online records of China’s flora (http://www.cn-flora.ac.cn/index.html accessed on 15 March 2023), it has a creeping rhizome. The rhizome is aromatic, with a thickness of 2–5 mm, externally light brown, and its internode length is 3–5 mm, with mostly fibrous roots. The rhizome’s upper part is very dense, and branches are often fibrous, persisting at the leaf base. Leaves are sessile with a thin leaf blade, with membranous leaf sheaths up to 5 mm wide on both sides of the base, ascending to the middle of the leaf blade, tapering, and undergoing abscission. The leaf blade is dark green, linear, 20–30 cm long, and its base is folded in half and spread above the middle. It is 7–13 mm wide, with a tapering apex, no middle rib, many parallel veins, and a slightly raised angle. It has an axillary inflorescence stalk, 4–15 cm long, that is triangular. The bracts are 13–25 cm long, the fleshy spikes are 2–5 times longer, and are subequal in length. The inflorescences are terete, 4–6.5 cm long, 4–7 mm thick, superficially acuminate, erect, or slightly curved. The flowers are white. The mature fruit is 7–8 cm long and up to 1 cm thick. The young fruits are green, yellow-green, or yellow-white when mature. The flowering period is from February to June. Usually, rhizomes are dug out in autumn and winter, and leaves and fibrous roots are removed, cleaned, and further dried to obtain the medicinal part of *A. tatarinowii*. The *A. tatarinowii* herbal parts used in Chinese medicine are usually flat or long and thick. The plant’s features are shown in Figure 1. The outer skin is gray-brown, with some visible links and root marks. The cut surface is fibrous, white, or reddish, with distinct rings and oil spots. It has a sweet odor and a bitter, pungent taste. The observation of some sections of *A. tatarinowii* under the microscope showed that the outer wall of epidermal cells on the transverse section of the *A. tatarinowii* rhizome was thickened and brown, and some also contained reddish-brown substances. The cortex of *A. tatarinowii* is wide, with scattered fiber bundles and leaf trace vascular bundles. The leaf trace vascular bundle is externally hardened, and the vascular bundle sheath fibers are ringed and lignified; the endodermis is clearly visible. The vascular bundle of the middle column is of the wood type and outer type, and the vascular bundle sheath fiber is less. The cells around the fiber bundles and vascular bundle sheath fibers contain calcium oxalate crystals, forming crystalline fibers. Round-like oil cells are scattered in parenchyma cells that contain starch granules.

## 3. Traditional Uses

*A. tatarinowii* has been widely used as a medicinal plant in China for 2000 years. Since ancient times, researchers have continuously explored and exploited TCM practices. TCM uses in the treatment and prevention of disease have boosted trust and resolve in its advancement and innovation. In the recorded history of folk culture, *A. tatarinowii* is a commonly used TCM. Generally speaking, each TCM has its inherent taste and characteristics. *A. tatarinowii* has a bitter and spicy taste and a warm nature. In addition, according to the different meridians of each TCM, *A. tatarinowii* has a stimulating effect on the heart and stomach meridians. Based on its action on these meridians, it can calm the mind, resolve dampness, harmonize the stomach, and unblock painful obstructions. It releases the exterior while dispersing cold and expelling wind-dampness. The property of sexual taste meridian attribution is very important in guiding clinical drug applications in the TCM system [14]. It is used for multiple medicinal purposes, traditionally for treating epilepsy, depression, fever, dizziness after a high fever, deafness, heartache, stomachache, and other diseases. *A. tatarinowii* has a long medicinal use history in China, and it is not only an important TCM itself but is also a critical part of TCM prescriptions [15,16,17,18,19]. In addition to using *A. tatarinowii* to treat different diseases, *A. tatarinowii* can be combined with different TCMs to achieve improved therapeutic effects. For example, *A. tatarinowii* is commonly used with TCMs such as bupleurum and turmeric, which have significant antidepressant effects, to prepare a mixed formulation to improve its antidepressant effect. It is commonly used in depression-like disorders in the clinical environment [20,21]. Further, preclinical studies have shown that *A. tatarinowii* has strong antidepressant activity. Many studies have found that its water extract, ethanol extract, and extract with other solvents have strong activity from the perspective of different extraction methods. Further studies have shown that the active ingredient asarone exhibits a strong antidepressant effect and is of great research value [9]. In addition to this, the *A. tatarinowii* ethanol extract has antifungal activity and can be used to treat digestive diseases, such as diarrhea [1]. In addition, in Korean medicine, after a lot of verification, it has also been found that *A. tatarinowii* has a positive therapeutic effect on brain diseases such as meningitis and is also effective for AD, Parkinson’s disease (PD), and other neurological diseases caused by population aging. In addition, the process of *A. tatarinowii* treating brain diseases and nervous system diseases has been found to be the same as in our cognition of the TCM system [22,23,24]. In short, the various therapeutic effects of *A. tatarinowii* in traditional uses, as well as its potential for future applications, have been supported by abundant evidence and warrant further investigation.

## 4. Phytochemistry

To date, *A. tatarinowii* has been investigated from a phytochemical perspective. The literature indicates the presence of numerous chemical compounds, such as phenylpropanoids, terpenoids, lignans, flavonoids, alkaloids, amides, organic acids, and other classes. Until now, more than 160 compounds have been isolated and characterized. These compounds are summarized in Table 1.

### 4.1. Phenylpropanoids

**Figure 2:**
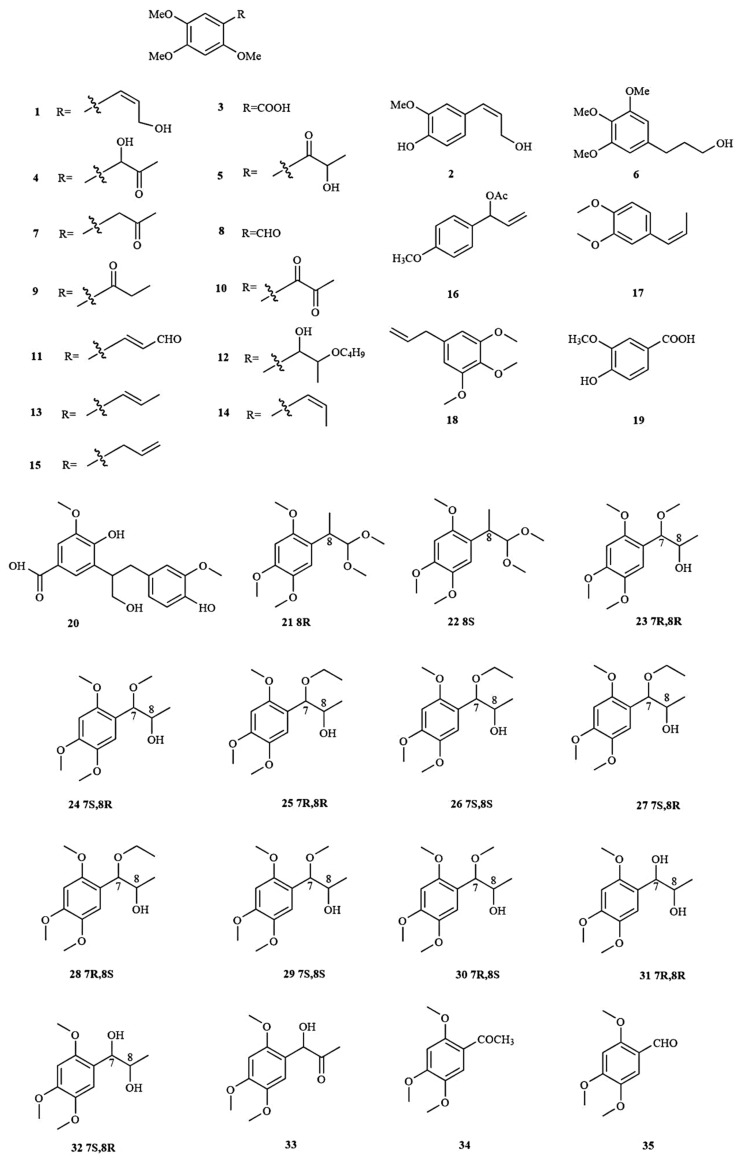
The structures of phenylpropanoids in *A. tatarinowii*.

Phenylpropanoids are a class of natural compounds that contain one or several C6-C3 units in their structure. Phenylpropanoids isolated from *A. tatarinowii* often have a specific characteristic structure, bearing methoxy groups in the benzene ring. Till now, 35 phenylpropanoids (**1**–**35**) have been isolated from the rhizomes of *A. tatarinowii* (Figure 2). Among the phenylpropanoids, α-asarone and β-asarone were reported to be the major *A. tatarinowii* constituents.

### 4.2. Sesquiterpenes

**Figure 3:**
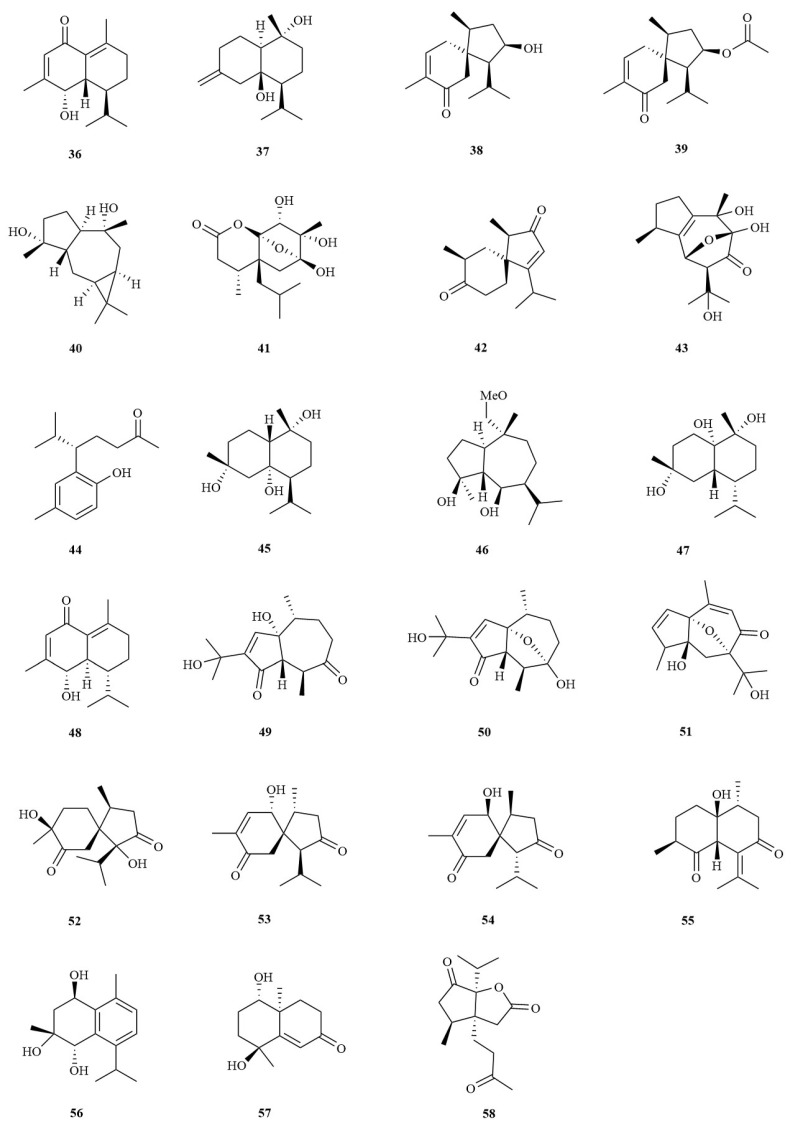
The structures of sesquiterpenes in *A. tatarinowii*.

At present, more than 20 sesquiterpenoids have been isolated from *A. tatarinowii*. Sesquiterpenes are the most abundant group of terpenoids, whose skeleton is composed of 3 isoprene units and contains 15 carbon atoms. The oxygenated derivatives of sesquiterpenes have a strong aroma and biological activity and are also important raw materials in the medicine, food, and cosmetics industries. Most sesquiterpenes in *A. tatarinowii* are monocyclic sesquiterpenes (Figure 3).

### 4.3. Lignans

**Figure 4:**
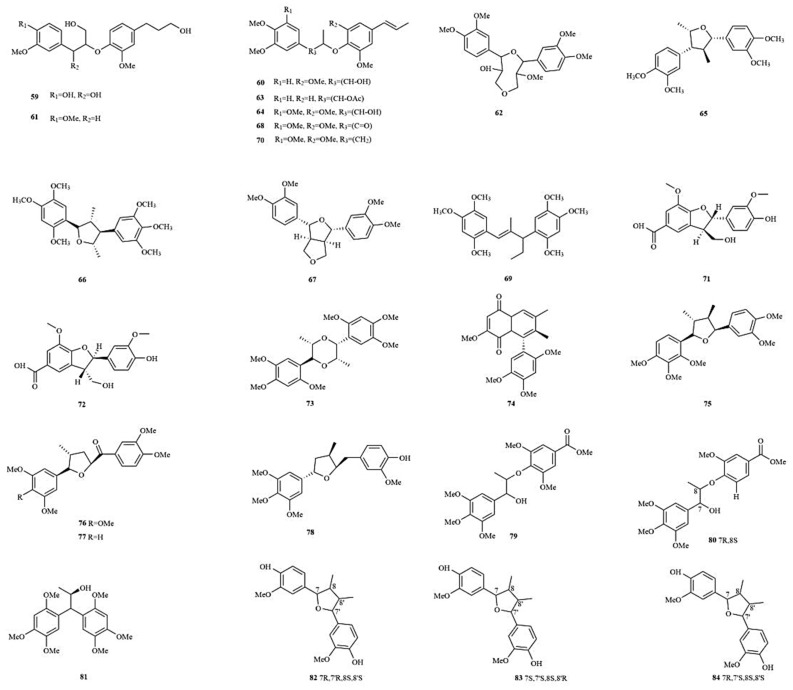

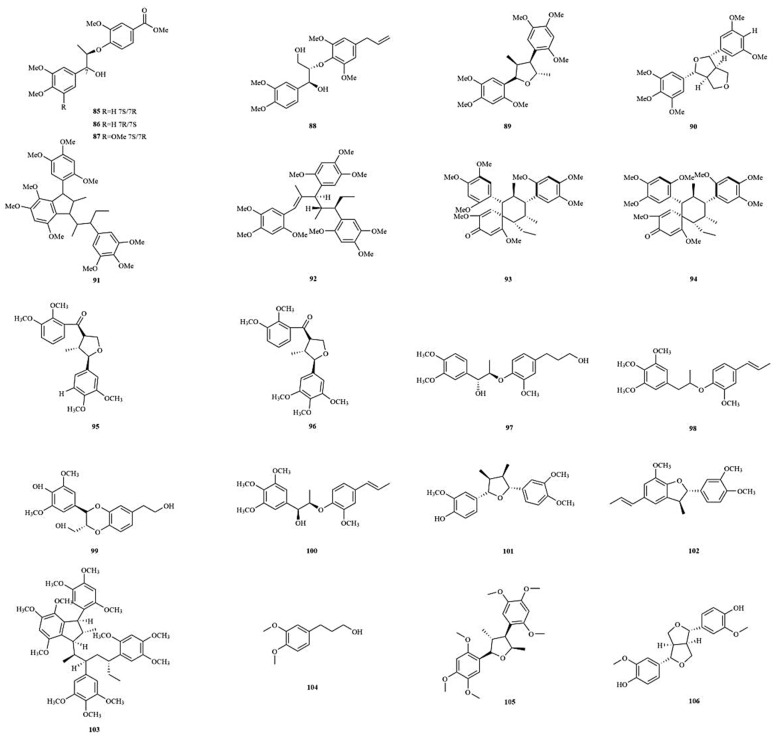
The structures of lignans in *A. tatarinowii*.

Lignans are a class of natural compounds that result from the polymerization of two molecules (a few are from three or four molecules) of phenylpropanoid derivatives, and they are mainly present in the wood and resin of plants. The lignan monomers are mainly composed of cinnamic acid, cinnamyl alcohol, propenyl benzene, and allyl benzene [44]. At present, more than 40 lignans have been extracted from *A. tatarinowii*, mainly divided into two structural types: monoepoxide lignans and double epoxide lignans. Among them, Veraguensin (**65**), Magnosalicin (**66**), (2*S*,3*R*)-ceplignan (**71**), (2*R*,3*S*)-ceplignan (**72**), Acortatarinowin I (**75**), Acortatarinowin J (**76**), Acortatarinowin K (**77**), Acortatarinowin L (**78**), Saucernetindiol (**82**), Machilin-I (**83**), and others are monoepoxide lignans, while Tatarinowin (**62**), Eudesmin (**67**), (±)-Acortatarinowin F (**90**), and (±)-Pinoresinol (**106**) are double epoxide lignans (Figure 4).

### 4.4. Flavonoids

**Figure 5:**
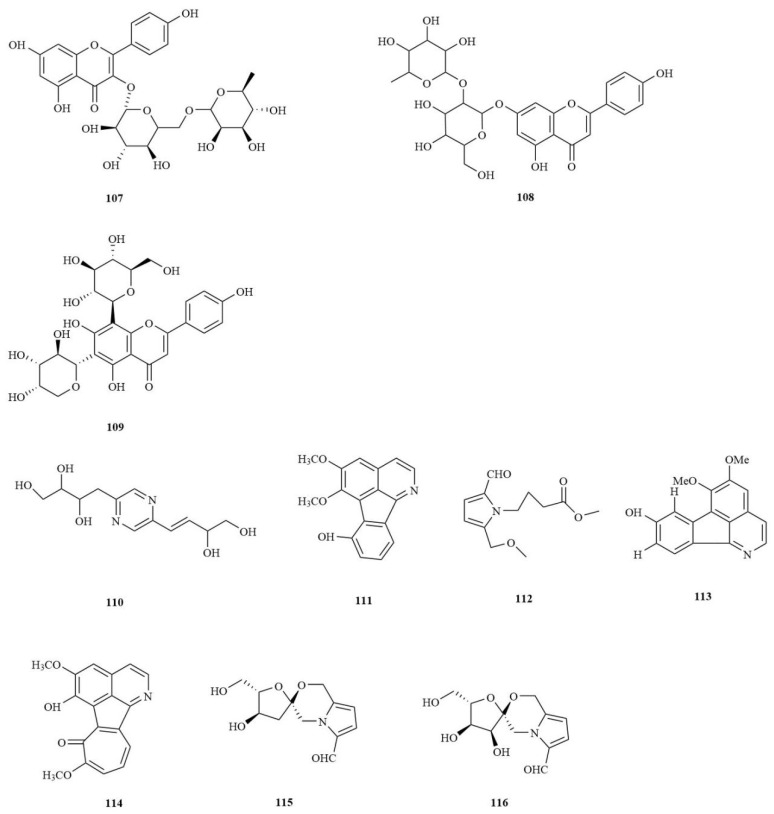
The structures of flavonoids and alkaloids in *A. tatarinowii*.

Flavonoids are a class of compounds with a core nucleus of a 2-phenyl chromone molecule and no oxygen-containing group substitution at the 3-position. They are widely present in the plant kingdom and are among the most active natural active ingredients [45]. Three flavonoid glycosides have been isolated from rhizomes of *A. tatarinowii*: Kaempferol-3-*O*-rutinoside (**107**), Rhoifolin (**108**), and Isoschaftoside (**109**). Compared with other compounds, there is not a lot of information on *A. tatarinowii* flavonoid structure. Therefore, future efforts should be made to isolate and characterize flavonoids in *A. tatarinowii*. The chemical structures of the flavonoid compounds are shown in Figure 5.

### 4.5. Alkaloids

Alkaloids are secondary metabolites that contain nitrogen atoms in the negative oxidation state and are present in biological organisms. They are alkaline organic compounds containing nitrogen. Alkaloids are also important plant chemical constituents known to have various pharmacological effects in humans and animals. To date, seven alkaloids (**110**–**116**) have been isolated from *A. tatarinowii* (Figure 5).

### 4.6. Amides

**Figure 6:**
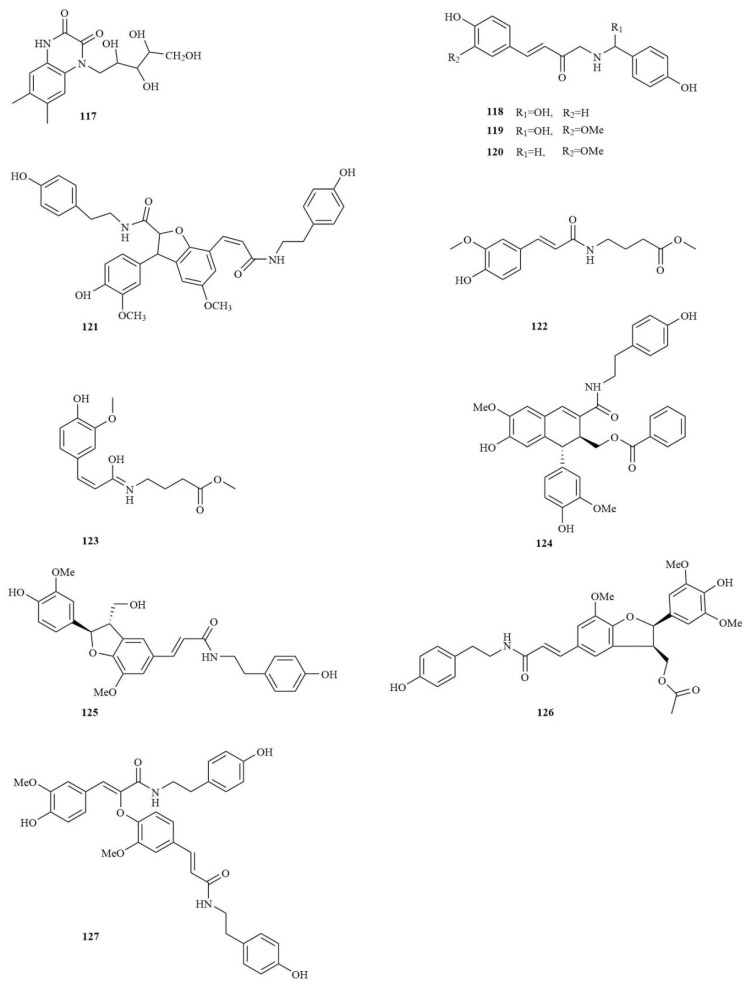
The structures of amides in *A. tatarinowii*.

Acyl compounds linked to nitrogen atoms are termed amides, a class of nitrogen-containing carboxylic acid derivatives. The amide bond is the most typical functional group in chemical, biological, and pharmaceutical compound synthesis. Because of the importance of amide bonds, their synthesis has become the most commonly used reaction in drug synthesis. Over 11 amides have been discovered in *A. tatarinowii* (**117**–**127**), which are derived from a straight chain amide with an isobutyl group (Figure 6).

### 4.7. Organic Acids

**Figure 7:**
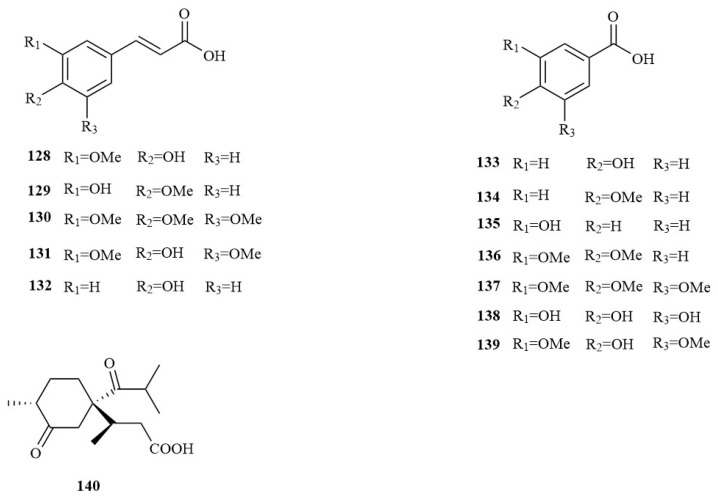
The structures of organic acids in *A. tatarinowii*.

Organic acids are a class of compounds containing carboxyl groups and are abundant in the leaves, roots, and especially fruits of plants. Most of them are present in the form of salt, and some of them are combined into esters. Thirteen organic acids were isolated from *A. tatarinowii*, of which **128**–**132** were aromatic organic acids. Most organic acids isolated from *A. tatarinowii* exhibit weak acidity (Figure 7).

### 4.8. Others

**Figure 8:**
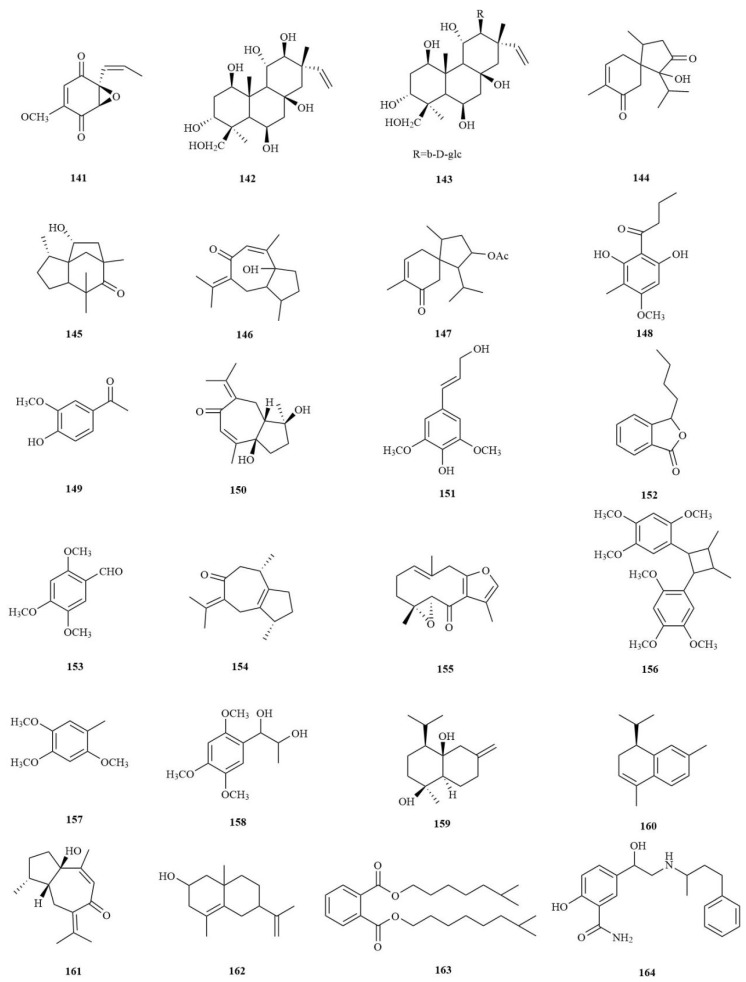
The structures of others compounds in *A. tatarinowii*.

In addition to the compound classes mentioned above, more than 20 other compounds have been isolated from *A. tatarinowii*, including the diterpenoids Tatarol (**142**) and Tataroside (**143**), the phenolic compounds Aspidinol (**148**) and Apocynin (**149**), the esters 3-butyl-phthalide (**152**) and diisocaprylphthalate (**163**), the ether polymer Bisasaricin (**156**), and others (Figure 8). The above findings illustrate the wide chemical composition of *A. tatarinowii*, which is of immense future research value.

## 5. Pharmacological Activities

**Figure 9:**
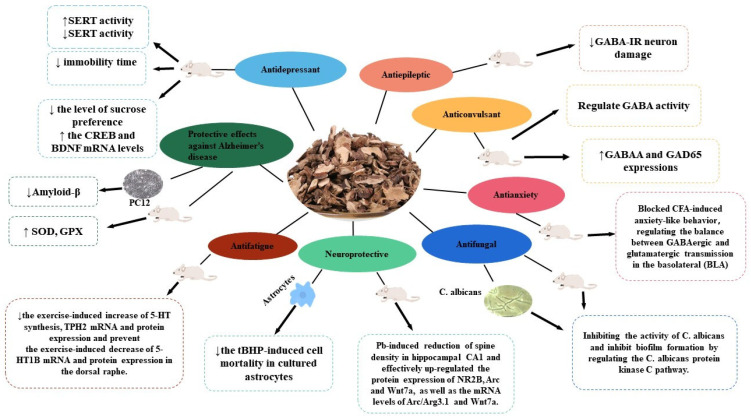
The pharmacological activities of *A. tatarinowii*. (“↑” indicates an upward revision; “↓” indicates a downward revision).

Modern pharmacological research has revealed that *A. tatarinowii* exerts various pharmacological activities, including antidepressant, antiepileptic, anticonvulsant, antianxiety, neuroprotective, antifatigue, antifungal, improving AD, and others. These increasingly in-depth pharmacological studies provide an improved scientific basis for clinical practice (Figure 9). The properties of *A. tatarinowii* active compounds, their pharmacological effects, and potential mechanisms of action on the basis of different types of extracts and compounds are summarized in Table 2.

### 5.1. Antidepressant Properties

Depression, under the increasing pressure of social activities, has gradually become one of the most common psychiatric disorders. It severely limits psychosocial functioning and quality of life. At the same time, it is becoming a heavy economic burden to society and families [6,56]. Studies have shown that the water extract of *A. tatarinowii* can be effective against depression. Experiments were carried out using the forced swimming test (FST), tail suspension test (TST), and locomotor activity (LA) in mice. The mice were acclimated in a quiet laboratory for 60 min and then placed in water alone for 6 min. The mice were suspended from the tail end with tape at about 2 cm from the tail tip so that the mice were suspended 15 cm from the ground. Their movement within the next 30 min was recorded using a high-definition digital camera. The immobility time of mice in the above conditions was recorded. The results confirmed that the *A. tatarinowii* water extracts significantly decreased mice immobility time but did not alter the mice’s locomotor activity. At the same time, the serotonin transporter (SERT) activity was significantly increased at a dose of 100 μg/mL of the *A. tatarinowii* water extract. Moreover, the petroleum ether extract of *A. tatarinowii* also significantly increased SERT activity at a dose of 1.56 μg/mL. In contrast, the water extract after petroleum ether processing significantly inhibited SERT activity at 50–100 μg/mL. Thus, *A. tatarinowii* could regulate SERT activities in a bidirectional manner, potentially exerting its antidepressant properties [9]. In addition, α-asarone and β-asarone, the main components of essential oil (EO) from the rhizome of *A. tatarinowii*, were found to exhibit antidepressant effects. The same experiment was carried out in mice, showing that at a 5, 10, and 20 mg/kg dose of α-asarone and β-asarone, the immobility time of mice was significantly reduced (*p* > 0.01). The antidepressant, imipramine, was the positive control at a dose of 15 mg/kg. Notably, α-asarone significantly reduced the immobility time at doses of 10 and 20 mg/kg (*p* > 0.05 and *p* > 0.01) compared with the control. Furthermore, the immobility time was also decreased by β-asarone at a dose of 20 mg/kg (*p* > 0.05). The mean immobility times after α-asarone and β-asarone administration were as follows: α-asarone (5, 10, and 20 mg/kg) 205.1 ± 19, 178 ± 15, and 159 ± 17 s, β-asarone (5, 10, and 20 mg/kg) 223 ± 23, 198 ± 18, and 179 ± 18 s. These results indicate dose-dependent antidepressive-like activities of α-asarone and β-asarone [46].

In addition, to evaluate the influence of the *A. tatarinowii* β-asarone on depressive-like behavior induced by the chronic unpredictable mild stress (CUMS) model, the CUMS rat model of depression was used. During 28 straight days at a volume of 0.01 g/mL, β-asarone (25 mg/kg/day) or an equivalent amount of saline served as the control for rats exposed to CUMS. When compared to CUMS-exposed rats, the time spent motionless was considerably decreased by 29% after being administered β-asarone (*p* < 0.05). Moreover, the Sucrose Preference Test (SPT) revealed that sucrose preference was 45% lower in CUMS-exposed rats as compared with non-stressed control rats. Additionally, β-asarone in *A. tatarinowii* was shown to promote hippocampal neuronal neurogenesis in CUMS-exposed rats, significantly increasing the CREB and BDNF mRNA levels. Furthermore, this research also showed that adult neurogenesis plays a role in the antidepressant-like behavioral outcomes of β-asarone, indicating that β-asarone from *A. tatarinowii* is a prospective option for the treatment of depression [47]. In conclusion, *A. tatarinowii* extracts can have a potent antidepressant effect, providing an important natural medicine option for treating depression.

### 5.2. Antiepileptic Properties

Epilepsy is a neurological disease caused by abnormal neuron discharge in the brain. Its onset often leads to temporary brain dysfunction, accompanied by fainting, convulsions, and other pathological reactions, affecting the normal life of many patients [57,58]. Studies have shown that *A. tatarinowii* extract has antiepileptic effects [59]. The maximal electroshock (MES), pentylenetetrazol (PTZ) maximal seizure, and prolonged PTZ kindling models were used to test the extract’s antiepileptic properties. Mice with persistent convulsions with tonic hindlimb extension were randomly divided into different groups. Electric stimulation was given 45 min after intraperitoneal administration of the drug or normal saline (NS), and each group’s convulsive rate was recorded. PTZ at 100 mg/kg was injected intraperitoneally 45 min after the drug or NS administration. Convulsive and mortality rates, as well as seizure latency, were recorded. The results indicated that both the decoction (at a dose of 10–20 g/kg) and volatile oil (at 1.25 g/kg) of *A. tatarinowii* significantly decreased the epileptic rate in the MES model. The *A. tatarinowii* decoction was effective in the PTZ model, with decreased epileptic and mortality rates. In the dosage range studied, the *A. tatarinowii* volatile oil was unable to prevent seizures, although a dose of 1.25 g/kg was observed to lengthen seizure latency and reduce mortality. The long-term PTZ kindling model was established in male Sprague Dawley (SD) rats. In the PTZ kindling model, γ—aminobutyric acid (GABA) immunohistochemical reaction (IR) (GABA-IR) neurons decreased significantly compared with the normal group. After therapy with the decoction and volatile oil, the severity of the seizures dramatically diminished in the treated groups. As compared to PTZ kindling controls, more GABA-IR neurons were discovered. Morphological examination also indicated that GABA-IR neuron loss was less severe in the drug-treated groups. All in all, both the decoction and volatile oil extracted from *A. tatarinowii* were shown to possess antiepileptic properties. The volatile oil was less effective for PTZ-induced epilepsies. Both extracts could prevent epileptic episodes, as well as epilepsy-related GABAergic neuron damage in the brain in the prolonged PTZ kindling model [48,60,61]. These results provide a scientific basis for the clinical antiepileptic application of *A. tatarinowii* and benefit the development and production of novel antiepileptic drugs.

### 5.3. Anticonvulsant Properties

The typical clinical manifestations of convulsive seizures are sudden loss of consciousness and sudden generalized or localized, tonic or clonic facial and limb muscle convulsions. Prolonged convulsions can cause hyperthermia, hypoxic brain damage, cerebral edema, and even cerebral hernia, which can be life-threatening [62,63]. *A. tatarinowii* lignans, especially eudesmin, have shown significant anticonvulsant effects on mice. MES- and PTZ-induced seizures in male mice were used to evaluate the anticonvulsant activities of eudesmin. Mice were pretreated with intraperitoneal injections of eudesmin (5, 10, and 20 mg/kg), while NS (20 kg/mL) was used as the blank control group. Mice from the MES model group were intraperitoneally injected with phenytoin (20 mg/kg) again, while the PTZ model group was injected with diazepam (4 mg/kg). After 30 min, the MES model group was given an electric shock, and the PTZ model group was subcutaneously injected with PTZ (90 mg/kg). The results showed that eudesmin exhibited significant anticonvulsant effects at the 5, 10, and 20 mg/kg doses. In addition, no convulsion or death was observed in the mice treated with the positive control drugs phenytoin 20 mg/kg and diazepam 4 mg/kg. Finally, the eudesmin mechanism of action was investigated by determining the glutamic acid (Glu) and gamma-aminobutyric acid (GABA) content in epileptic mice and glutamate decarboxylase 65 (GAD65), GABAA, Bcl-2, and caspase-3 gene expression in the brain of chronic epileptic rats. The MES and PTZ test results revealed that eudesmin isolated from *A. tatarinowii* possesses significant anticonvulsant effects. Furthermore, after eudesmin treatment, the GABA content increased, whereas the Glu content decreased, and the ratio of Glu/GABA decreased. Moreover, GAD65, GABAA, and Bcl-2 were up-regulated after treatment with eudesmin, whereas caspase-3 was down-regulated. In summary, the anticonvulsant effect of eudesmin isolated from *A. tatarinowii* may be associated with the up-regulation of GABAA and GAD65 expression and neuron anti-apoptosis in the brain [49,64]. Another study found that the anticonvulsant effect against the pain models in mice was observed when an *A. tatarinowii* methanol extract was administered orally at 100 and 200 mg/kg doses. The anticonvulsant effect was studied through the PTZ-induced seizures method. The results suggest that the activity of GABA might potentiate the anticonvulsant effects [50]. In summary, these findings may provide novel directions or insights into treating convulsions using TCM, such as *A. tatarinowii.*

### 5.4. Antianxiety Properties

One of the most prevalent mental diseases and the primary contributor to psychosocial dysfunction is anxiety. This disorder causes high costs in terms of healthcare use, disability, loss of productivity, and patient quality of life [65]. A frequent comorbidity of chronic pain is anxiety illness. Those who experience chronic pain are more likely to develop anxiety problems, according to earlier research. Tian et al. discovered that *A. tatarinowii* extract has potential antianxiety properties. Previous studies have shown that anxiety-like behavior could be induced in mice with persistent inflammatory pain. First, to induce chronic inflammatory pain, a single dose of complete Freund’s adjuvant (CFA) (50% of 10 μL CFA) was injected into the plantar surface of the left hind paw. Mice exhibited significant anxiety-like behavior two weeks after the CFA injection [66]. The mice were placed in a central square device, allowing free movement for 5 min. The number of entries and time spent in each treatment arm were recorded. Mice were placed in the box’s center and allowed to explore freely for 15 min. A decrease in the time spent and the number of entries in the open arms in the elevated plus maze test (EPMT) revealed the anxiety-related behavioral phenotype as well as a decrease in the percentage of time spent in central areas in the open field test (OFT). Administration of α-asarone (2 and 20 mg/kg) for one week (from day 8 to day 14 after CFA injection) inhibited the anxiety-like behavior in a dose-dependent manner without affecting locomotor activity. This was achieved by regulating the balance between GABAergic and glutamatergic transmission in the basolateral (BLA), achieving partially inhibited chronic pain-induced anxiety-like behaviors in mice [51,67]. On the other hand, it has been suggested that GABAergic inhibition is essential for the modulation and maintenance of excitation/inhibition balance. GABAA receptors play the most important role in GABAergic inhibition. Clinically, some anxiolytic drugs exert their effects by binding with the GABAA receptors [68,69]. The above investigations will serve as a guide for more in-depth clinical uses of *A. tatarinowii* for the treatment of anxiety.

### 5.5. Neuroprotective Properties

At present, there are many protective mechanisms for neurological disorders, and oxidative stress in the neuronal cell has been proposed to play a crucial role in disease progression [70]. The use of *A. tatarinowii* and its primary components, α-asarone and β-asarone, in treating neurological illnesses, particularly in neuroprotection, has been supported by a number of lines of evidence [71,72,73,74]. Test-butyl hydroperoxide (tBHP)-induced rat primary astrocytes were used to evaluate the neuroprotective properties of the volatile oil and asarone from *A. tatarinowii*. Primary cultured rat astrocytes were plated and pretreated with different medications for 48 h. Then, the cultures were treated with tBHP for 3 h. Cultured rat astrocytes were pretreated with α-asarone, β-asarone, or the *A. tatarinowii* volatile oil for 48 h. The *A. tatarinowii* representative constituents exhibited promising protective effects on the cultures. The administration of tBHP in the cultures led to the induction of oxidative stress and cell death, with the application of tBHP considerably lowering cell viability in a dose-dependent manner. The application of these *A. tatarinowii* representative constituents protected against cell death induced by the tBHP challenge. The tBHP-induced cell death in the cultured astrocytes was considerably decreased after a dose-dependent pretreatment. The *A. tatarinowii* representative constituents did not show cytotoxicity nor a proliferating effect on the cultures in all the concentrations (0.5 to 15 μg/mL) applied. In addition, α-asarone and β-asarone (3, 10, and 30 mg/kg) have demonstrated antioxidant effects in several animal seizure models [75,76]. They perform a crucial protective function in maintaining normal levels of superoxide dismutase, lipid peroxidation, catalase, and glutathione-peroxidase in various stressed-out areas of rat brains. This suggests that they play a neuroprotective role through an antioxidant pathway [52,77].

Moreover, the β-asarone of *A. tatarinowii* exhibited neuroprotective effects against spatial memory impairment and synaptogenesis in the chronic lead (Pb)-exposed rats. Both SD developmental rat pups and adult rats were used in the study. Rat pups were exposed to Pb throughout the lactation period, and β-asarone (10 and 40 mg/kg) was given intraperitoneally from postnatal day 14 to 21. In addition, the adult rats were exposed to Pb from the embryo stage to 11 weeks old, and β-asarone (2.5, 10, and 40 mg/kg) was given during the period from 9 to 11 weeks old. The Morris water maze test and Golgi–Cox staining method were used to assess spatial memory ability and synaptogenesis. Rats were anesthetized with CO_2_ and quickly decapitated. The brains were longitudinally cut into two halves. One hemisphere was processed for morphological staining, and the other hemisphere was used to examine specific protein expression. It should be noted that *A. tatarinowii* constituents can pass through the blood–brain barrier quickly [78,79]. It effectively attenuated the Pb-induced reduction of spine density in hippocampal CA1 and dentate gyrus areas in a dose-dependent manner both in developmental and adult rats. At the same time, the Pb-induced impairments of learning and memory were partially rescued. In addition, it resulted in the up-regulation of NR2B, Arc, and Wnt7a protein expression, as well as an increase in the mRNA levels of Arc/Arg3.1 and Wnt7a [12]. In conclusion, the neuroprotective properties of *A. tatarinowii* offer an intriguing treatment strategy for a variety of neurological disorders.

### 5.6. Protective Effects against Alzheimer’s Disease

AD is a degenerative disease of the central nervous system primarily characterized by the progressive loss of cognition and memory. AD has several pathological hallmarks, including extracellular amyloid plaque formation, intracellular neurofibrillary tangles, and neuronal loss [80,81,82]. The most important feature of AD is the gradual, irreversible cognitive ability loss through amyloid β (Aβ) plaque formation and of neurofibrillary tangles composed of tau protein [83]. Previous studies have shown that this TCM has ameliorative and protective properties against neurodegenerative diseases, such as Parkinson’s disease and AD, hypoxic–ischemic encephalopathy, and cerebrovascular diseases [84].

β-asarone, the main *A. tatarinowii* constituent, plays an important role in the central nervous system. Wang et al. established the AD cell model, culturing PC12 cells *in vitro*, and Aβ_1–42_ was then added into the medium at different concentrations and time points. As the concentration of Aβ_1–42_ and time increased, the PC12 cell viability decreased in a dose-dependent manner; at the same time, cytotoxicity and LDH increased. Moreover, senescent cells clearly increased in cells treated with Aβ_1–42_. After establishing a stable AD cell model, they investigated the effects of gradient concentrations of β-asarone (12, 24, 36, 72, and 144 μM) or donepezil (10, 20, and 40 μM). The β-asarone protective effect on cell proliferation was dose-dependent; the low-dose group demonstrated a better protective effect than the high-dose group. Subsequently, 24, 36, and 72 μM of β-asarone and 9.6 μM of donepezil were chosen as the ideal concentrations, respectively. Compared with model cells, β-asarone and donepezil both improved cell proliferation and decreased cell damage [85,86]. At the same time, they also decreased the cell senescence rate. In conclusion, the study demonstrated that the β-asarone in *A. tatarinowii* can inhibit Aβ, which has a significant therapeutic effect against toxic protein deposition [54,87].

Another study used adult male Wistar rats to examine the effects of β-asarone on neurodegeneration brought on by intrahippocampal injection of Aβ. The Alzheimer’s disease model was established, and then the rats were treated with β-asarone (12.5, 25, and 50 mg/kg). Rats were randomly divided into groups and were bilaterally injected with Aβ. Thirty days before Aβ administration, an intragastric tube was used to administer β-asarone for fifty days, every day. Once the rats were sacrificed, the hippocampal homogenate’s oxidative stress parameters, superoxide dismutase (SOD), and glutathione peroxidase (GPX) activity were assessed. The results showed that β-asarone at doses of 25 and 50 mg/kg significantly increased the levels of antioxidant enzymes, including SOD and GPX. Moreover, β-asarone significantly decreased cell loss in the cerebral cortex and hippocampus [55,88,89]. These findings suggest that *A. tatarinowii* and its active constituent β-asarone have potential therapeutic effects against Alzheimer’s disease, which could be useful for the development of new drugs.

### 5.7. Antifatigue Properties

Fatigue may be defined as the inability to maintain the expected muscle strength, leading to reduced performance during prolonged exercise. However, the cause is usually not muscle fatigue but an increase in serotonin or 5-hydroxytryptamine (5-HT) concentration in the brain during prolonged exercise [90,91]. *A. tatarinowii* is an ancient TCM tonic nourishment that can be used as an antifatigue medicine. The influence of *A. tatarinowii* on endurance exercise was determined by the fatigue time of adult male rats during a treadmill exercise. Rats were injected with *A. tatarinowii* water extract (1, 10, and 100 mg/kg) two hours before the treadmill exercise. Caffeine was used as the positive control drug. *A. tatarinowii* prolonged the time to exhaustion by treadmill exercise in a dose-dependent way. Notably, *A. tatarinowii* at 100 mg/kg was just as effective as caffeine (10 mg/kg) in prolonging the time to exhaustion during the treadmill exercise. By preventing the exercise-induced decrease in 5-HT1B mRNA and protein expression in the dorsal raphe, *A. tatarinowii* was able to increase exercise endurance. It could also attenuate the exercise-induced increase in 5-HT synthesis, the TPH2 mRNA and protein expression, and other effects. Moreover, the effects of *A. tatarinowii* were comparable to those of caffeine [56,92]. These findings support the traditional medical application of *A. tatarinowii* and point to its potential therapeutic value as an antifatigue drug.

### 5.8. Antifungal Properties

Fungal infections can result in many diseases, including dermatosis with skin infections and fungal enteritis with acute or chronic infections of deep tissues, causing significant morbidity and mortality in susceptible populations. *Candida* spp. are common opportunistic fungal pathogens, among which *Candida albicans* is the most common infectious fungal agent [93,94]. *C. albicans* is a normal human intestinal, oral cavity, and vaginal microflora constituent. It can cause infections ranging from easily treatable superficial infections to life-threatening invasive infections [95,96]. The ethanol extract of *A. tatarinowii* was shown to possess antifungal activities *in vivo* and *in vitro*. Its fungicidal efficiency was evaluated in vivo, with mice randomly divided into four groups. In the first group, mice were pricked with a needle in their abdomens and orally fed PBS as the KB-negative control group. The other three groups of mice were infected intraperitoneally with 5 × 10^5^ CFU of log-phase *C. albicans*. After two days, mice of groups 2–4 were orally fed with the ethanol extract of *A. tatarinowii*, fluconazole (positive drug control group), or PBS (negative control group) (8 mg/kg) once every day for seven days. After seven days, the ethanol extract of *A. tatarinowii* significantly reduced the fungal burden in the spleen, liver, and kidney compared to fluconazole. These results suggest that ethanol extract of *A. tatarinowii* can be used to treat deep *C. albicans* infections [97]. Additionally, a sterile filter paper disk impregnated with ethanol extract of *A. tatarinowii* was placed on an agar plate, inoculated with a *C. albicans* suspension, and incubated under aerobic conditions for 24 h. The diameters of inhibition zones were then measured and recorded. *A. tatarinowii* resulted in an inhibition zone of 9.9 ± 0.5 mm against *C. albicans*, compared with 7 mm in the control group. Further, the MIC and MFC values of *A. tatarinowii* against *C. albicans* were 51.2 and 102.4 μg/mL. *A. tatarinowii* showed significantly higher potency against *C. albicans* than the two positive control drugs, fluconazole and itraconazole, at 51.2 μg/mL [1]. In summary, the ethanol extract of *A. tatarinowii* has superior antifungal activity in vivo and in vitro [98]. These results could contribute to reducing antibiotic consumption for the treatment of fungal infections, thereby helping to reduce the emergence of antibiotic resistance. They further promote the safe and effective use of *A. tatarinowii* for traditional and modern medical applications.

## 6. Conclusions and Perspectives

**Figure 10:**
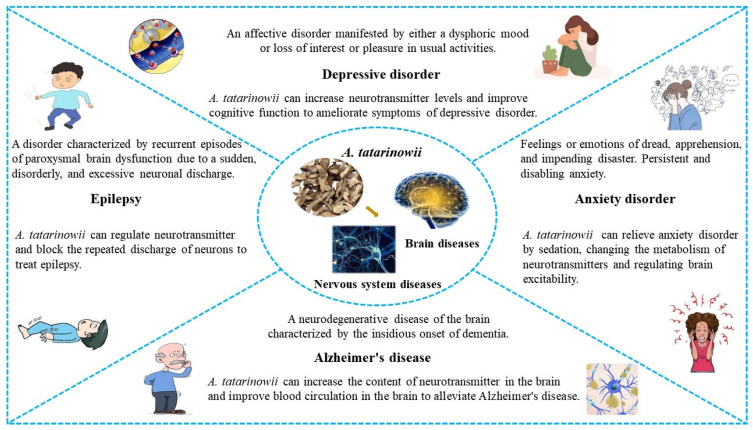
Therapeutic effects of *A. tatarinowii* on brain diseases and nervous system diseases.

This review provided the scientific foundation for future research on *A. tatarinowii* and the development of better therapeutic agents using the natural medicinal plant. At the same time, according to the traditional literature and contemporary evidence, the present research status of *A. tatarinowii* was critically reviewed. Nowadays, *A. tatarinowii* is widely used in the treatment of brain diseases and nervous system diseases and has achieved satisfactory therapeutic effects (Figure 10). *A. tatarinowii* can treat brain diseases, such as epilepsy, anxiety, and depression, by regulating neurotransmitter levels. It can also improve blood circulation in the brain to alleviate neurological diseases, such as AD. To date, over 160 compounds have been isolated and identified from *A. tatarinowii*. It is expected that more active ingredients will be identified and characterized in future research. On the other hand, pharmacological studies published in the literature with in vitro and in vivo assays largely corroborate its wide medicinal use. These studies indicated that both the extracts and active constituents of *A. tatarinowii* possess a wide range of pharmacological activities. These modern pharmacological studies supported most traditional uses of *A. tatarinowii* as an indispensable TCM.

Although significant work has been conducted on *A. tatarinowii*, some scientific gaps still need to be explored. Firstly, the reported studies have shown that the main chemical components of *A. tatarinowii* are phenylpropanoids. At the same time, there are relatively few other chemical constituents extracted and isolated from *A. tatarinowii*. More chemical constituents must be identified to explore the relationship between bioactive constituents and pharmacological effects in depth. More advanced instruments to separate and identify rare compounds in *A. tatarinowii* should be utilized to study their pharmacological activity. The active components of the aboveground parts of *A. tatarinowii*, such as stems and leaves, should be studied to contribute to the rational utilization of the plant’s resources and to identify the concentration of active compounds in the different plant tissues. Secondly, the research on the medicinal parts of *A. tatarinowii* is not comprehensive enough. Due to the highly variable secondary metabolism in plants, the chemical components and pharmacological effects of the different medicinal parts of *A. tatarinowii* plants are also very different. *A. tatarinowii* showed good anti-AD properties. Due to the aging society, the morbidity and prevalence rate of senile diseases such as AD and PD are growing at an accelerated pace. Therefore, it is urgent to study a new and novel drug for treating AD. The active ingredients of *A. tatarinowii* come from different tissues of the plant. Further pharmacological studies should be conducted on different chemical components of rhizomes and/or any other parts of *A. tatarinowii* to provide a sufficient scientific basis and in-depth research on the function and mechanism of the identified active ingredients. We expect this will be the key direction of future research. Thirdly, systematic data on pharmacokinetics and clinical studies of *A. tatarinowii* are limited, and there are few studies on target organ toxicity. We should conduct more clinical studies to evaluate possible therapeutic effects and investigate the side effects and toxicity of *A. tatarinowii*. The toxic effect of asarone in *A. tatarinowii* may limit its therapeutic effect. Toxicological studies have shown that α-asarone and β-asarone in *A. tatarinowii* can cause hepatomas, which may have mutagenic, genotoxic, and teratogenic effects. It has been reported that β-asarone is more toxic than α-asarone. In a study involving the human body, several consumers experienced persistent vomiting due to long-term intake of *A. tatarinowii* containing high concentrations of asarone. In addition, asarone also showed cytotoxic and genotoxic effects on HepG2 cells. Due to the potential toxic effects of asarone, in particular β-asarone, the European Council has limited the content of β-asarone in alcoholic beverages and condiments to 1 mg/kg and in other foods and beverages to 0.1 mg/kg. Based on the existing literature, further dose-dependent in vivo studies are needed to confirm the mutagenicity, genotoxicity, and teratogenicity associated with α-asarone and β-asarone. In addition, it is speculated that the epoxide metabolites of α-asarone and β-asarone may be the cause of these toxicities. Importantly, considering the toxicity of α-asarone and β-asarone, the clinical use of these compounds carries certain risks. On the other hand, other compounds such as sesquiterpene Acorusin E and indole alkaloids in *A. tatarinowii* have also been reported to have potential toxicity. Studies have shown that high doses of Acorusin E in *A. tatarinowii* may inhibit the central nervous system. Secondly, long-term or high-dose intake of indole alkaloids in *A. tatarinowii* may have some adverse effects on the human body, causing nausea, vomiting, dizziness, diarrhea, and other symptoms. In addition, indole alkaloids may also have effects on the cardiovascular system, such as arrhythmia and blood pressure changes [7,99,100]. Therefore, it is necessary to be cautious in determining the human administration regimen of *A. tatarinowii* to avoid toxicity and protect human health. Further research may focus on the pharmacodynamic material relationship, pharmacokinetics, clinical research, and toxicological evaluation of *A. tatarinowii*. At the same time, *A. tatarinowii* has potential as a nutritional supplement that promotes health. As people are increasingly conscious of their well-being, there is a growing demand for edible Chinese medicines that offer health benefits. Thus, further studies should be conducted on *A. tatarinowii* health products to explore their potential for future development. The premise of in-depth development and utilization of natural plant resources may be evaluating and controlling their quality. However, there are still some shortcomings in the quality control of *A. tatarinowii*. Nowadays, *A. tatarinowii* on the market is easy to confuse with *Anemone altaica* Fisch. Fortunately, they belong to different families of plants or are easy to distinguish. In addition, since the TCM composition and pharmacological effects are usually complex, for their quality control, single components and multiple components may be utilized to assess the quality of a specific TCM, especially the chemical components related to its efficacy. The study of the pharmacodynamic material basis of *A. tatarinowii* mostly focuses on the study of its volatile components. According to the latest edition of the pharmacopeia (2020), the volatile oil content in the chemical composition of *A. tatarinowii* should not be less than 1.0% (mL/g), and the volatile oil in the Chinese herbal pieces should not be less than 0.7% (mL/g) [4]. However, the volatile oil components of *A. tatarinowii* are mixtures and unstable. With further in-depth research on *A. tatarinowii*, certain unique components of *A. tatarinowii,* such as phenylpropanoids and lignans, can be selected as its quality markers [101,102]. This will provide a solid foundation for scientifically developing and utilizing more *A. tatarinowii* plant resources. This will also help to protect people’s health and safety better. Therefore, it is necessary to develop new and effective analytical methods and techniques to identify multiple components to achieve more comprehensive quality control of *A. tatarinowii*.

In summary, *A. tatarinowii* is an important medicinal plant and a source of phytochemicals with extensive pharmacological activities and high application value in all respects. However, further comprehensive and in-depth clinical studies are required to determine the safety and availability of *A. tatarinowii* for clinical utility. Until now, many compounds from *A. tatarinowii* have been found, but further research needs be conducted to provide a more thorough characterization. In the future, the structure–activity relationship and mechanistic action of isolated compounds should be studied to explore their potency and drug-like properties. The present paper systematically reviews the botany, traditional uses, phytochemistry, and pharmacology of *A. tatarinowii*. We aimed to provide the groundwork for further research on its mechanism of action and the development of improved therapeutic agents using *A. tatarinowii* in the future. Furthermore, we hope this review highlights the importance of *A. tatarinowii* and provides useful directions for the future development of this natural medicinal plant.

## Figures and Tables

**Table 1:** Chemical compounds isolated from *A. tatarinowii*.

No.	Chemical Component	Molecular Formula	Extraction Solvent	Plant Parts	Reference
Phenylpropanoids					
1	Acoramo	C_12_H_16_O_4_	MeOH	Rhizomes	[25]
2	(*Z*)-Coniferyl alcohol	C_10_H_12_O_3_	MeOH	Rhizomes	[25]
3	2,4,5-trimethoxybenzoic acid	C_10_H_12_O_5_	MeOH	Rhizomes	[25]
4	Tatarinoids B	C_12_H_16_O_5_	MeOH	Rhizomes	[25]
5	Tatarinoids A	C_12_H_16_O_5_	MeOH	Rhizomes	[25]
6	3-(3,4,5-trimethoxyphenyl) propan-1-ol	C_12_H_18_O_4_	MeOH	Rhizomes	[25]
7	Acoramone or isoacoramone	C_12_H_16_O_4_	MeOH	Rhizomes	[25]
8	Asaronaldehyde	C_10_H_12_O_4_	MeOH	Rhizomes	[25]
9	Isoacoramone or acoramone	C_12_H_16_O_4_	MeOH	Rhizomes	[25]
10	1-(2,4,5-trimethoxyphenyl) propan-1,2-dione	C_12_H_14_O_5_	MeOH	Rhizomes	[25]
11	(*E*)-3-(2,4,5- trimethoxyphenyl) acrylaldehyde	C_12_H_14_O_4_	MeOH	Rhizomes	[25]
12	2,4,5-trimethoxyl-2′-butoxy-1,2-phenyl propandiol	C_16_H_26_O_5_	MeOH	Rhizomes	[25]
13	α-Asarone	C_12_H_16_O_3_	MeOH	Rhizomes	[25]
14	β-Asarone	C_12_H_16_O_3_	MeOH	Rhizomes	[25]
15	γ-Asarone	C_12_H_16_O_3_	MeOH	Rhizomes	[25]
16	1-(4-methoxyphenyl) allyl acetate	C_12_H_14_O_3_	MeOH	Rhizomes	[25]
17	*Cis*-methylisoeugenol	C_11_H_14_O_2_	MeOH	Rhizomes	[26]
18	Elemicin	C_12_H_16_O_2_	MeOH	Rhizomes	[26]
19	Benzoic acid	C_8_H_8_O_4_	95% EtOH	Rhizomes	[1]
20	(*R*)-4-hydroxy-3-[1-hydroxy-3-(4-hydroxy-3-methoxyphenyl)propan-2-yl]-5-methoxyben-zoic acid	C_18_H_20_O_7_	Water	Rhizomes	[27]
21	(*R*)-1-(1,1-dimethoxypropan-2-yl)-2,4,5-trimethoxybenzene [(−)-*R*-isoacorphenylpropanoid]	C_14_H_22_O_5_	60% EtOH	Rhizomes	[28]
22	(*S*)-1-(1,1-dimethoxypropan-2-yl)-2,4,5-trimethoxybenzene [(+)-*S*-isoacorphenylpropanoid]	C_14_H_22_O_5_	60% EtOH	Rhizomes	[28]
23	(7*R*,8*R*)-7-methoxy-8-hydroxy-dihydroasarone (ent-acoraminol A)	C_13_H_20_O_5_	60% EtOH	Rhizomes	[28]
24	(7*S*,8*R*)-7-methoxy-8-hydroxydihydroasarone (ent-acoraminol B)	C_13_H_20_O_5_	60% EtOH	Rhizomes	[28]
25	(7*R*,8*R*)-7-ethoxy-8-hydroxydihydroasarone (ent-acoraminol C)	C_14_H_22_O_5_	60% EtOH	Rhizomes	[28]
26	(7*S*,8*S*)-7-ethoxy-8-hydroxydihydroasarone(acoraminol C)	C_14_H_22_O_5_	60% EtOH	Rhizomes	[28]
27	(7*S*,8*R*)-7-ethoxy-8-hydroxy-dihydroasarone (ent-acoraminol D)	C_14_H_22_O_5_	60% EtOH	Rhizomes	[28]
28	(7*R*,8*S*)-7-ethoxy-8-hydroxydihydroasarone (acoraminol D)	C_14_H_22_O_5_	60% EtOH	Rhizomes	[28]
29	Acoraminol A	C_13_H_20_O_5_	60% EtOH	Rhizomes	[28]
30	Acoraminol B	C_13_H_20_O_5_	60% EtOH	Rhizomes	[28]
31	(7*R*,8*R*)-7,8-dihydroxydihydroa-sarone	C_12_H_18_O_5_	60% EtOH	Rhizomes	[28]
32	(7*S*,8*R*)-7,8-dihydroxydihydroa-sarone	C_12_H_18_O_5_	60% EtOH	Rhizomes	[28]
33	1-hydroxy-1-(2,4,5-trimethoxyphenyl)propan-2-one	C_12_H_16_O_5_	60% EtOH	Rhizomes	[28]
34	1-(2,4,5-trimethoxyphenyl)ethanone	C_11_H_14_O_4_	60% EtOH	Rhizomes	[28]
35	Asaraldehyde	C_10_H_12_O_4_	60% EtOH	Rhizomes	[28]
Sesquiterpenes					
36	2-oxocadinan-1(10),3-dien-5-ol	C_15_H_22_O_2_	Water	Rhizomes	[29]
37	Isocalamediol	C_15_H_26_O_2_	Water	Rhizomes	[29]
38	2-hydroxyacorenone	C_15_H_24_O_2_	Water	Rhizomes	[29]
39	2-acetoxyacorenone	C_17_H_26_O_3_	Water	Rhizomes	[29]
40	4a,10a-aroma-dendranediol	C_15_H_26_O_2_	Water	Rhizomes	[29]
41	6,7,8-trihydroxy-4a-isobutyl-4,7-dimethylhexahydro-6,8a-epoxychromen-2(3*H*)-one	C_15_H_24_O_6_	Water	Rhizomes	[27]
42	Acorusin D	C_15_H_22_O_2_	95% EtOH	Rhizomes	[30]
43	Acorusin E	C_15_H_22_O_5_	95% EtOH	Rhizomes	[30]
44	Litseachromolaevane A	C_15_H_22_O_2_	95% EtOH	Rhizomes	[30]
45	1β,7α(*H*)-cadinane- 4α,6α,10α-triol	C_15_H_28_O_3_	95% EtOH	Roots	[31]
46	1α,5β-guaiane-10α-*O*-ethyl- 4β,6β-diol	C_17_H_32_O_3_	95% EtOH	Rhizomes	[31]
47	6β,7β(*H*)-cadinane-1α,4α, 10α-triol	C_15_H_28_O_3_	95% EtOH	Rhizomes	[31]
48	Tatarinowin A	C_15_H_22_O_2_	Water	Rhizomes	[32]
49	Calamusin A	C_15_H_22_O_4_	95% EtOH	Rhizomes	[33]
50	Calamusin B	C_15_H_22_O_4_	95% EtOH	Rhizomes	[33]
51	Calamusin C	C_15_H_20_O_4_	95% EtOH	Rhizomes	[33]
52	Calamusin D	C_15_H_24_O_4_	95% EtOH	Rhizomes	[33]
53	Calamusin E	C_15_H_22_O_3_	95% EtOH	Rhizomes	[33]
54	Calamusin F	C_15_H_22_O_3_	95% EtOH	Rhizomes	[33]
55	Calamusin G	C_15_H_22_O_3_	95% EtOH	Rhizomes	[33]
56	Calamusin H	C_15_H_22_O_3_	95% EtOH	Rhizomes	[33]
57	Calamusin I	C_12_H_18_O_3_	95% EtOH	Rhizomes	[33]
58	Neo-acorane A	C_15_H_22_O_4_	95% EtOH	Rhizomes	[34]
Lignans					
59	4,7,9,9′-tetrahydroxy-3,3′-dimethoxy-8-*O*-4′- neolignan	C_20_H_26_O_7_	MeOH	Rhizomes	[25]
60	(7*R*,8*R*)-Virolin	C_22_H_28_O_6_	MeOH	Rhizomes	[25]
61	Ligraminol D	C_21_H_28_O_6_	MeOH	Rhizomes	[25]
62	Tatarinowin	C_23_H_30_O_8_	MeOH	Rhizomes	[25]
63	Ligraminol C	C_23_H_28_O_6_	MeOH	Rhizomes	[25]
64	Polysphorin	C_23_H_30_O_6_	MeOH	Rhizomes	[25]
65	Veraguensin	C_22_H_28_O_5_	MeOH	Rhizomes	[25]
66	Magnosalicin	C_24_H_32_O_7_	MeOH	Rhizomes	[25]
67	Eudesmin	C_22_H_26_O_6_	MeOH	Rhizomes	[25]
68	2*S*-(2,6-dimethoxy-4- propenyl-phenoxy)-1 - (3,4,5-trimethoxy-phenyl)- propane-1-one	C_23_H_28_O_7_	MeOH	Rhizomes	[25]
69	Diasarone I	C_24_H_32_O_6_	MeOH	Rhizomes	[25]
70	1,3-dimethoxy-2-[1-methyl-2-(3,4,5-trimethoxyphenyl)- ethoxy]-5-(1- propenyl-1-yl)-benzen	C_23_H_30_O_6_	MeOH	Rhizomes	[25]
71	(2*S*,3*R*)-ceplignan	C_18_H_20_O_7_	Water	Rhizomes	[27]
72	(2*R*,3*S*)-ceplignan	C_18_H_20_O_7_	Water	Rhizomes	[27]
73	Acortatarinowin G	C_24_H_32_O_8_	95% EtOH	Rhizomes	[35]
74	Acortatarinowin H	C_22_H_24_O_6_	95% EtOH	Rhizomes	[35]
75	Acortatarinowin I	C_23_H_30_O_6_	95% EtOH	Rhizomes	[35]
76	Acortatarinowin J	C_23_H_28_O_7_	95% EtOH	Rhizomes	[35]
77	Acortatarinowin K	C_22_H_26_O_6_	95% EtOH	Rhizomes	[35]
78	Acortatarinowin L	C_22_H_28_O_6_	95% EtOH	Rhizomes	[35]
79	Acortatarinowin M	C_22_H_28_O_9_	95% EtOH	Rhizomes	[35]
80	Acortatarinowin N	C_21_H_26_O_8_	95% EtOH	Rhizomes	[35]
81	Tatarinoid C	C_21_H_28_O_7_	95% EtOH	Rhizomes	[35]
82	Saucernetindiol	C_20_H_24_O_5_	95% EtOH	Rhizomes	[35]
83	Machilin-I	C_20_H_24_O_5_	95% EtOH	Rhizomes	[35]
84	Verrucosn	C_20_H_24_O_5_	95% EtOH	Rhizomes	[35]
85	(±)-Acortatarinowin A	C_20_H_24_O_7_	95% MeOH	Rhizomes	[36]
86	(±)-Acortatarinowin B	C_20_H_24_O_7_	95% MeOH	Rhizomes	[36]
87	(±)-Acortatarinowin C	C_21_H_26_O_8_	95% MeOH	Rhizomes	[36]
88	(±)-Acortatarinowin D	C_20_H_24_O_7_	95% MeOH	Rhizomes	[36]
89	(±)-Acortatarinowin E	C_22_H_28_O_7_	95% MeOH	Rhizomes	[36]
90	(±)-Acortatarinowin F	C_23_H_28_O_7_	95% MeOH	Rhizomes	[36]
91	Tatarinan O	C_36_H_48_O_9_	95% EtOH	Roots	[37]
92	Tatanan A	C_36_H_48_O_9_	95% EtOH	Rhizomes	[38]
93	Tatanan B	C_35_H_46_O_9_	95% EtOH	Rhizomes	[38]
94	Tatanan C	C_35_H_46_O_9_	95% EtOH	Rhizomes	[38]
95	Tatarinoid D	C_22_H_26_O_6_	60% EtOH	Rhizomes	[39]
96	Tatarinoid E	C_23_H_28_O_7_	60% EtOH	Rhizomes	[39]
97	Tatarinoid F	C_21_H_28_O_6_	60% EtOH	Rhizomes	[39]
98	Tatarinoid G	C_22_H_28_O_5_	60% EtOH	Rhizomes	[39]
99	Tatarinoid H	C_19_H_22_O_7_	60% EtOH	Rhizomes	[39]
100	[*S*,*R*-(*E*)]-3,4,5-trimethoxy-[1-[2-methoxy-4-(1-propenyl)phenoxy]ethyl]-benzenemethanol	C_22_H_28_O_6_	60% EtOH	Rhizomes	[39]
101	Nectandrin A	C_21_H_26_O_5_	60% EtOH	Rhizomes	[39]
102	(2*R*,3*R*)-2-(3,4-dimethoxyphenyl)-2,3-dihydro-7-methoxy-3-methyl-5-(1*E*)-1-propen-1-yl-benzofuran	C_21_H_24_O_4_	60% EtOH	Rhizomes	[39]
103	Tatarinan T	C_48_H_64_O_12_	95% EtOH	Roots	[40]
104	3-(3,4-dimethoxyphenyl)propan-1-ol	C_11_H_16_O_3_	60% EtOH	Rhizomes	[28]
105	(±)-Magnosalicin	C_24_H_32_O_7_	60% EtOH	Rhizomes	[28]
106	(±)-Pinoresinol	C_20_H_22_O_6_	60% EtOH	Rhizomes	[28]
Flavonoids					
107	Kaempferol-3-*O*- rutinoside	C_27_H_30_O_15_	MeOH	Rhizomes	[25]
108	Rhoifolin	C_27_H_30_O_14_	MeOH	Rhizomes	[25]
109	Isoschaftoside	C_26_H_28_O_14_	MeOH	Rhizomes	[25]
Alkaloids					
110	2-(3′,4′-dihydroxy-1′ -butylenyl)-5-(2″,3″,4″-trihydroxybutyl)-pyrazine	C_12_H_18_N_2_O_5_	Water	Rhizomes	[29]
111	Tatarine A	C_17_H_13_NO_3_	Water	Rhizomes	[29]
112	4-(2-formyl-5-methoxymethyl pyrrol- 1-yl)butyric acid methyl ester	C_12_H_17_NO_4_	Water	Rhizomes	[29]
113	Tatarine D	C_17_H_13_NO_3_	60% EtOH	Rhizomes	[41]
114	Neotatarine	C_18_H_13_NO_4_	95% EtOH	Rhizomes	[42]
115	Acortatarin A	C_12_H_15_NO_5_	Water	Rhizomes	[43]
116	Acortatarin B	C_12_H_15_NO_6_	Water	Rhizomes	[43]
Amides					
117	Tatarine C	C_15_H_20_N_2_O_6_	MeOH	Rhizomes	[25]
118	Tataramide A	C_17_H_17_NO_4_	MeOH	Rhizomes	[25]
119	(*S*)-*N*-trans-feruloyloctopamine	C_18_H_19_NO_5_	MeOH	Rhizomes	[25]
120	*N*-trans-Feruloyl-tyramine	C_18_H_19_NO_4_	MeOH	Rhizomes	[25]
121	Tataramide B	C_36_H_36_N_2_O_8_	MeOH	Rhizomes	[25]
122	(*E*)-methyl 4-[3-(4-hydroxy-3-methoxyphenyl) acrylamido]butanoate	C_15_H_19_NO_5_	Water	Rhizomes	[27]
123	(*Z*)-methyl-4-[3-(4-hydroxy-3-methoxyphenyl)acrylamido]butanoate enol isomer	C_15_H_21_NO_5_	Water	Rhizomes	[27]
124	Acorusin A	C_35_H_33_NO_8_	95% EtOH	Rhizomes	[30]
125	Grossamide K	C_28_H_29_NO_7_	95% EtOH	Rhizomes	[30]
126	Tatarine E	C_31_H_33_NO_9_	60% EtOH	Rhizomes	[41]
127	Cannabisin F	C_36_H_36_N_2_O_8_	60% EtOH	Rhizomes	[41]
Organic acids					
128	Ferulic acid	C_10_H_10_O_4_	Water	Roots and Rhizomes	[9]
129	Trans-Isoferulic acid	C_10_H_10_O_4_	Water	Roots and Rhizomes	[9]
130	3,4,5-trimethoxycinnamic acid	C_12_H_14_O_5_	Water	Roots and Rhizomes	[9]
131	3,5-dimethoxy-4-hydroxycinnamic acid	C_11_H_12_O_5_	Water	Roots and Rhizomes	[9]
132	Trans-4-hydroxycinnamic acid	C_9_H_8_O_3_	Water	Roots and Rhizomes	[9]
133	4-hydroxybenzoic acid	C_7_H_6_O_3_	Water	Roots and Rhizomes	[9]
134	Anisic acid	C_8_H_8_O_3_	Water	Roots and Rhizomes	[9]
135	3-hydroxybenzoic acid	C_7_H_6_O_3_	Water	Roots and Rhizomes	[9]
136	Veratric acid	C_9_H_10_O_4_	Water	Roots and Rhizomes	[9]
137	3,4,5-trimethoxybenzoic acid	C_10_H_12_O_5_	Water	Roots and Rhizomes	[9]
138	Gallic acid	C_7_H_6_O_5_	Water	Roots and Rhizomes	[9]
139	Syringic acid	C_9_H_10_O_5_	Water	Roots and Rhizomes	[9]
140	Acoric acid	C_15_H_24_O_4_	95% EtOH	Rhizomes	[34]
Other types					
141	1-*cis*-propenyl-1*S*,6*R*- epoxy-4-methoxy- 2,5-quinone	C_10_H_10_O_4_	MeOH	Rhizomes	[25]
142	Tatarol	C_20_H_34_O_7_	MeOH	Rhizomes	[25]
143	Tataroside	C_26_H_44_O_12_	MeOH	Rhizomes	[25]
144	Isocalamediol	C_15_H_22_O_3_	MeOH	Rhizomes	[25]
145	Calamensesquiterpinenol	C_15_H_24_O_2_	MeOH	Rhizomes	[25]
146	2,3,3a,7,8,8a-hexahydro-3a-hydroxy-1,4-dimethyl- 7-(1-methylethylidene)- 6(1*H*)-azulenone	C_15_H_22_O_2_	MeOH	Rhizomes	[25]
147	2-acetoxyacorenone	C_17_H_26_O_3_	MeOH	Rhizomes	[25]
148	Aspidinol	C_12_H_16_O_4_	95% EtOH	Rhizomes	[1]
149	Apocynin	C_9_H_10_O_3_	95% EtOH	Rhizomes	[1]
150	Aeru-gidiol	C_15_H_22_O_3_	95% EtOH	Rhizomes	[1]
151	Ethanone	C_11_H_14_O_4_	95% EtOH	Rhizomes	[1]
152	3-butyl-phthalide	C_12_H_14_O_2_	95% EtOH	Rhizomes	[1]
153	Asaraldehyde	C_10_H_12_O_4_	95% EtOH	Rhizomes	[1]
154	Cala-musenone	C_15_H_22_O	95% EtOH	Rhizomes	[1]
155	Zederone	C_15_H_18_O_3_	95% EtOH	Rhizomes	[1]
156	Bisasaricin	C_24_H_32_O_6_	95% EtOH	Rhizomes	[1]
157	3,4,5-trimethoxytoluene	C_10_H_14_O_3_	95% EtOH	Rhizomes	[1]
158	1-(2,4,5-trimethoxyphenyl)-1,2-propanediol	C_12_H_18_O_5_	95% EtOH	Rhizomes	[1]
159	Calamendiol	C_15_H_26_O_2_	95% EtOH	Rhizomes	[1]
160	α-calacorene	C_15_H_20_	95% EtOH	Rhizomes	[1]
161	Acotatarone C	C_15_H_22_O_2_	95% EtOH	Rhizomes	[1]
162	Cyperol	C_15_H_24_O	95% EtOH	Rhizomes	[1]
163	Diisocapryl phthalate	C_24_H_38_O_4_	95% EtOH	Rhizomes	[1]
164	Labetalol	C_19_H_24_N_2_O_3_	95% EtOH	Rhizomes	[1]

**Table 2:** Summary of pharmacological activities of *A. tatarinowii* extracts/compounds.

Pharmacological Activities	Study Design	Models	Results/Mechanisms	Dosages	Reference
Antidepressant	*In vivo*	Male C57/BL6 mice	↑SERT activity ↓SERT activity	1.56 μg/mL 50–100 μg/mL	[9]
	*In vivo*	Male ICR mice	Significantly reduced immobility time	5, 10, and 20 mg/kg	[46]
	*In vivo*	CUMS rats	Significantly reduced immobility time, reduced the level of sucrose preference, and increased the CREB and BDNF mRNA levels	25 mg/kg	[47]
Antiepileptic	*In vivo*	Kunming mice and male SD rats	↓GABA-IR neuron damage	100 mg/kg	[48]
Anticonvulsant	*In vivo*	Male ICR mice or SD rats	Up-regulation of GABA_A_ and GAD65 expressions and anti-apoptosis of neurons in the brain	5, 10, and 20 mg/kg	[49]
	*In vivo*	The pain models in mice	Regulate GABA activity	100 and 200 mg/kg	[50]
Antianxiety	*In vivo*	Mice (chronic inflammatory mouse model)	Blocked CFA-induced anxiety-like behavior, regulating the balance between GABAergic and glutamatergic transmission in the basolateral (BLA)	20 mg/kg	[51]
Neuroprotective	*In vitro*	SD rats in cultured astrocytes	The tBHP-induced cell mortality in cultured astrocytes was markedly reduced	0.5–15 μg/mL	[52]
	*In vivo*	Both SD developmental rat pups and adult rats	Pb-induced reduction of spine density in hippocampal CA1 and effectively up-regulated the protein expression of NR2B, Arc, and Wnt7a, as well as the mRNA levels of Arc/Arg3.1 and Wnt7a	(10, 40 mg/kg) and (2.5, 10, 40 mg/kg)	[12]
Protective effects against Alzheimer’s disease	*In vitro*	PC12 cell	Inhibits Amyloid-β	12, 24, 36, 72, and 144 μM	[53]
	*In vivo*	Male Wistar rats	Significantly increased the levels of antioxidant enzymes, including SOD and GPX	12.5, 25, and 50 mg/kg	[54]
Antifatigue	*In vivo*	Adult male SD rats	Suppress the exercise-induced increase in 5-HT synthesis, TPH2 mRNA, and protein expression and prevent the exercise-induced decrease in 5-HT1B mRNA and protein expression in the dorsal raphe	100 mg/kg	[55]
Antifungal	*In vivo* and *in vitro*	Six-week-old female Kunming mice and *C. albicans* strain	Inhibiting the activity of *C. albicans* and inhibit biofilm formation by regulating the *C. albicans* protein kinase C pathway.	8 mg/kg	[1]

“↑” indicates a upward revision; “↓” indicates a downward revision.

## Data Availability

Not applicable.
